# Computational and Experimental Investigation of Chiral and Achiral Two‐Dimensional Organic Lead Bromide Perovskites: Octahedral Distortions and Electronic and Optical Properties

**DOI:** 10.1002/cphc.202500423

**Published:** 2025-10-27

**Authors:** Md Mehdi Masud, Jarek Viera, Azza Ben‐Akacha, Biwu Ma, David A. Strubbe

**Affiliations:** ^1^ Department of Physics University of California, Merced Merced CA 95343 USA; ^2^ Department of Chemistry and Biochemistry Florida State University Tallahassee FL 32306 USA

**Keywords:** chirality, density functional calculations, perovskite phases, UV/vis spectroscopy

## Abstract

A computational investigation is presented, in conjunction with synthesis and experimental characterization, into the structural, electronic, and optical properties of layered two‐dimensional organic lead bromide perovskites. Materials based on the chiral (R/S)‐4‐fluoro‐α‐methylbenzylammonium (R/S‐FMBA), which have been shown to lead to bright room‐temperature circularly polarized luminescence, are contrasted with the similar achiral 4‐fluorobenzylammonium (FBA). Using density functional theory (DFT) with van der Waals (vdW) corrections, relaxed structures (compared with X‐ray diffraction, XRD) and optical absorption spectra (compared with experiments) are studied, as well as band structure and orbital character of transitions. A Python code is developed and provided to calculate octahedral distortions and compare DFT and XRD results, finding that vdW corrections are important for accuracy and that DFT overestimates octahedral tilt angles. (FMBA)_2_PbBr_4_ shows among the largest tilt angle differences (often termed Δβ) reported, 14°–15°, indicating strong inversion symmetry‐breaking, which enables its chiral emission. A large resulting Dresselhaus spin‐splitting effect is found. The lowest‐energy optical transitions involve the perovskite only and are polarized within the layer. This work furthers understanding of structure‐property relations with applications to optoelectronics and spintronics.

## Graphical abstract

Octahedral distortion parameters in two‐dimensional hybrid organic‐inorganic perovskites, as calculated by Python code, are correlated with electronic and optical properties. Structure, Dresselhaus spin splitting, and anisotropic optical transitions of synthesized chiral and achiral lead bromide perovskites are computationally analyzed.

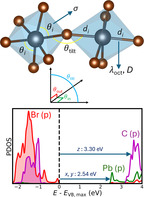

## 1 Introduction

Two‐dimensional (2D) organic‐inorganic hybrid perovskites (OIHPs)^[^ ^1^, ^2^, ^3^ ^]^ have garnered significant attention due to their unique optoelectronic properties, structural versatility, and potential for chiroptoelectronic and spintronic applications.^[^ ^3^, ^4^, ^5^ ^]^ These materials are characterized by their ability to incorporate chiral organic ligands,^[^ ^6^ ^]^ which impart chirality to the inorganic sublattice, enabling applications in circularly polarized light detection, optical information processing, and spin‐selective devices. Of particular interest are the chiral 2D OIHPs such as (R)‐ and (S)‐4‐fluoro‐α‐methylbenzylammonium (FMBA)‐based lead bromide perovskites, (R/S‐FMBA)_2_PbBr_4_.^[^ ^7^ ^]^ These materials have demonstrated promising properties, including room‐temperature circularly polarized luminescence and enhanced quantum yields when crystallized into oriented films, as shown by recent experimental work.^[^ ^7^ ^]^

Despite advances in synthesizing and characterizing these materials, understanding of their structural and electronic properties at the atomic level remains limited. Experimentally observed phenomena such as distortions in [PbBr_6_]^4−^ octahedra, hydrogen bonding effects, and their influence on electronic band structure and optical transitions are still not fully understood. The importance of octahedral distortions in perovskites has been appreciated for some time.^[^ ^8^, ^9^ ^]^ Studies in organic‐inorganic perovskites have shown that larger tilt angles between octahedra usually indicate more exciton trapping and wider bandgap.^[^ ^10^, ^11^ ^]^ Out‐of‐plane distortions have been established as a descriptor in 2D perovskites that is correlated with broadband emission.^[^ ^12^ ^]^ More recently, the deviation among tilt angles of different pairs of octahedra has been identified as a metric of inversion symmetry breaking in chiral perovskites, correlated with Rashba/Dresselhaus spin splittings.^[^ ^13^ ^]^ Pols et al. studied vector chirality‐based measures of chirality of the organic cations and in‐plane and out‐of‐plane aspects of the inorganic layers of 2D perovskites, as well as hydrogen bond asymmetry.^[^ ^14^ ^]^ Chirality has also been assessed for 2D perovskites via the “continuous chirality measure,” which parametrizes the deviations from inversion symmetry and was found to correlate with circular dichroism.^[^ ^15^ ^]^ Specifically for FMBA materials, the previous studies^[^ ^7^ ^]^ highlighted significant out‐of‐plane distortions in the inorganic layer and studied distortions within octahedra, but did not quantify octahedral tilting or fully connect structural distortions to their electronic and optical behaviors.

To gain further insight into structure‐property relationships, we synthesized (R/S‐FMBA)_2_PbBr_4_ materials experimentally and measured their circular dichroism to establish chirality, and also synthesized a related achiral reference material 4‐fluorobenzylammonium (FBA)_2_PbBr_4_. We then carried out a comprehensive computational study, using density functional theory (DFT) with van der Waals corrections to provide an in‐depth understanding of the behavior of these materials. We investigated structural relaxation and octahedral distortion parameters, with comparison to the X‐ray diffraction (XRD) structure. We studied electronic band structure, atomic orbital contributions, spin splitting, and polarized optical absorption spectra, compared with measured UV/vis absorption spectra.

We developed a Python code to easily calculate octahedral distortion parameters, adapting previous formulae^[^ ^8^, ^9^, ^12^, ^13^, ^16^ ^]^ and the MATLAB code from ref. [12] which calculates in‐plane and out‐of‐plane distortion angles. Other existing software includes Octadist^[^ ^17^ ^]^ and the visualization code VESTA,^[^ ^18^ ^]^ which can both calculate some octahedral distortion parameters. Our code brings together the calculation of a variety of intra‐ and inter‐octahedral distortion parameters in a single tool, and works in Python, which is free, widely available, and increasingly commonly used. This tool, included in the Supplementary Information, enables the quantification of bond length deviations and bond angle distortions for understanding structure‐property relationships in perovskite materials for optoelectronic and spintronics applications.

## 2 Experimental and Computational Methods

To investigate the structural, electronic, and optical properties of layered 2D lead bromide perovskites, we experimentally synthesized the materials and performed comprehensive DFT and random‐phase approximation (RPA) calculations to compare with the experimental data.

### 2.1 Experimental Synthesis and Characterization

**Figure 1:**
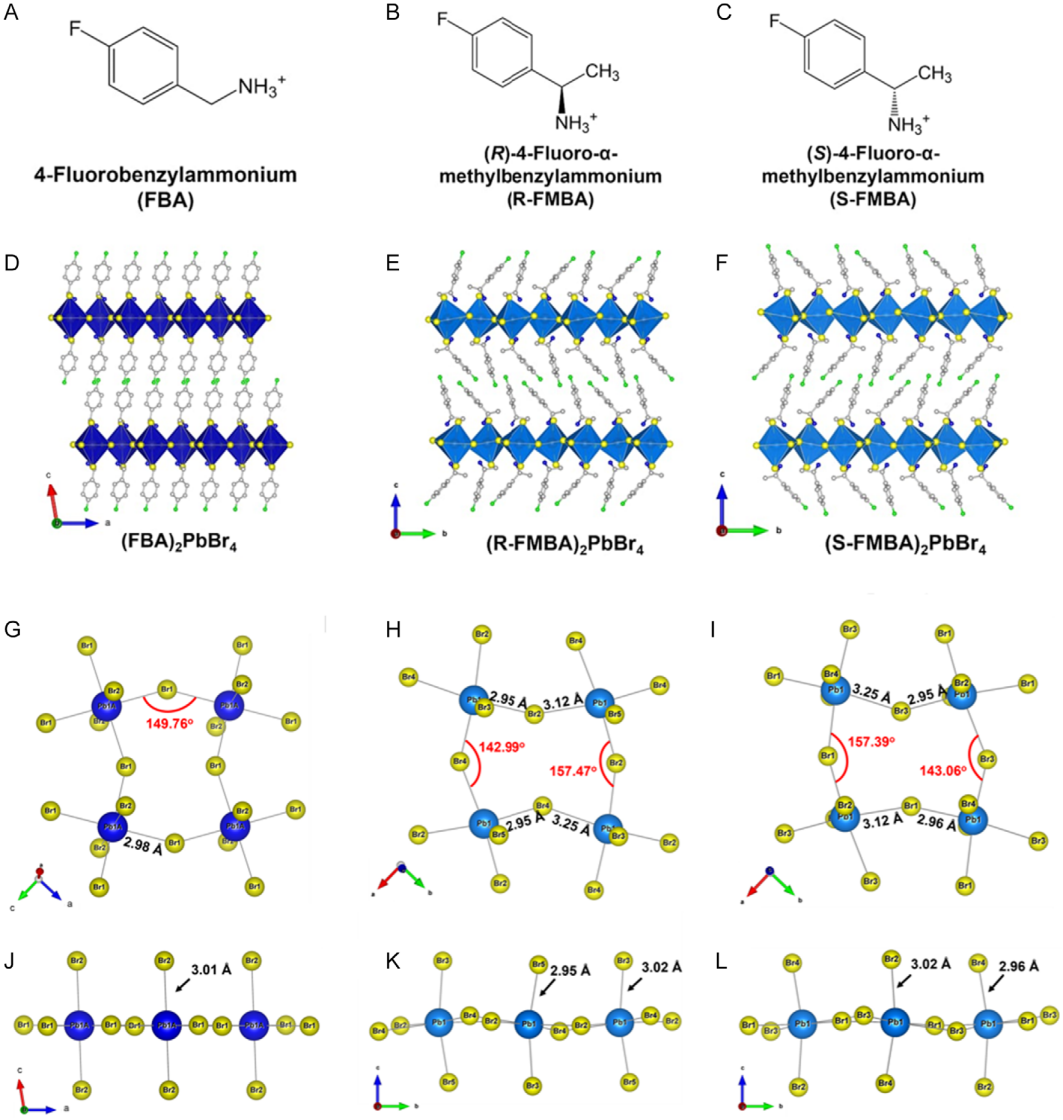
A–C) Organic ammonium cations, D–F) single‐crystal XRD structures of (FBA)_2_PbBr_4_, (R‐FMBA)_2_PbBr_4_, and (S‐FMBA)_2_PbBr_4_, G–I) top‐down view and J–L) side view of the connected lead bromide octahedra with different bond angles and lengths from the crystal structures of (FBA)_2_PbBr_4_, (R‐FMBA)_2_PbBr_4_, and (S‐FMBA)_2_PbBr_4_.

We synthesized three layered 2D perovskites: (R‐FMBA)_2_PbBr_4_, (S‐FMBA)_2_PbBr_4_, and (FBA)_2_PbBr_4_. Briefly, we prepared these perovskites by reacting the respective hydrobromide salts of the organic cations with PbBr_2_ in hydrobromic acid at 100 °C. Upon cooling, transparent plate‐like crystals grew from solution. All three crystals form the same general layered‐2D framework, composed of ${\text{PbBr}}_{4}^{2-}$ sheets separated by two adjacent organic cation layers, though the R‐ and S‐forms adopt chiral crystal structures in the orthorhombic space group *P*2_1_2_1_2_1_, with two layers per unit cell, while the FBA compound is achiral, crystallizing in the monoclinic *P*2_1_/*c* centrosymmetric space group with only one layer per unit cell (**Figure** **1**). The structure of (FBA)_2_PbBr_4_ consists of 41 atoms in the formula unit and 82 atoms in the unit cell. In contrast, the (R/S‐FMBA)_2_PbBr_4_ structures each contain 47 atoms in the formula unit and 188 atoms in the unit cell. Their single‐crystal X‐ray diffraction (SCXRD) data confirm that all three exhibit layered‐2D arrangements of corner‐sharing PbBr_6_ octahedra. The phase purity of the crystals and their uniformity were also confirmed by powder XRD. Further details of the synthesis and characterization procedures can be found in the Supplementary Information, as well as the XRD structures in crystallographic information file (CIF) format. Note that two versions are provided for FBA, a first one used for analysis of the structure in this work and as a starting point for DFT relaxations, and a second one that was further refined to confirm accuracy. This refined structure was very similar, though showing out‐of‐plane disorder in Pb positions, and its checkCIF report is included as well in Supplementary Information.

### 2.2 DFT Calculations

Plane‐wave DFT calculations were performed using Quantum ESPRESSO version 7.0.^[^ ^19^ ^]^ The Perdew‐Burke‐Ernzerhof (PBE) exchange correlation‐functional^[^ ^20^ ^]^ was used, along with the Grimme D2 dispersion correction^[^ ^21^ ^]^ to account for van der Waals interactions. Optimized norm‐conserving Vanderbilt (ONCV) pseudopotentials,^[^ ^22^ ^]^ sourced from PseudoDojo,^[^ ^23^ ^]^ were used to describe the interactions between core and valence electrons. These calculations were performed without the spin‐orbit coupling (SOC) effect. It is often found in hybrid perovskites that the Kohn‐Sham bandgap estimate from a semilocal functional, such as PBE, is comparable to the true bandgap due to cancelation of an opening of the gap by quasiparticle effects and a closing of the gap by SOC,^[^ ^24^, ^25^, ^26^ ^]^ whereas the Kohn‐Sham bandgap with SOC is significantly underestimated. We separately performed calculations with SOC to calculate Rashba/Dresselhaus spin‐splitting effects, using fully relativistic pseudopotentials from PseudoDojo (ONCVPSP v0.4).

The kinetic energy cut‐off for the wavefunction was set to 1225 eV, and a 3 × 3 × 1 unshifted *k*‐grid was utilized for self‐consistent field (SCF) calculations. Convergence thresholds for forces and stresses were set to 10^−4^ Ry bohr^−1^ and 0.1 kbar, respectively. For density of states (DOS) calculations, a denser 6×6×2 unshifted *k*‐grid and a Gaussian 0.05 eV broadening were employed.

Variable cell relaxations were performed starting from the experimental XRD structures, leading to lattice parameters closely agreeing with the experimental values (see **Table** **1**). Importantly, the space groups of the structures were preserved during the relaxation process. The DFT‐relaxed structures are provided in Supplementary Information in CIF format.

**Table 1:** Relaxed lattice parameters for (FBA)_2_PbBr_4_ and (R/S‐FMBA)_2_PbBr_4_ calculated using DFT with and without van der Waals corrections, compared to experimental lattice parameters from our synthesized materials and from literature.^[^ ^7^ ^]^

Compound	Method	*a* [Å]	*b* [Å]	*c* [Å]	*α* [°]	*β* [°]	*γ* [°]
(FBA)_2_PbBr_4_ space group: P 2_1_/c	XRD [this work]	8.11	8.15	17.55	90.00	99.74	90.00
	DFT [PBE + vdW]	8.14	8.03	16.41	90.00	99.44	90.00
	DFT [PBE, no vdW]	8.31	8.26	18.65	90.00	102.00	90.00
(R‐FMBA)_2_PbBr_4_ space group: P 2_1_2_1_2_1_	XRD^[^ ^7^ ^]^	7.84	8.80	33.43	90.00	90.00	90.00
	XRD [this work]	7.88	8.82	33.78	90.00	90.00	90.00
	DFT [PBE + vdW]	7.76	8.68	32.48	90.00	90.00	90.00
(S‐FMBA)_2_PbBr_4_ space group: P 2_1_2_1_2_1_	XRD^[^ ^7^ ^]^	7.84	8.80	33.43	90.00	90.00	90.00
	XRD [this work]	7.88	8.82	33.80	90.00	90.00	90.00
	DFT [PBE + vdW]	7.77	8.68	32.45	90.00	90.00	90.00

Optical absorption spectra were computed at the RPA level using the BerkeleyGW code.^[^ ^27^ ^]^ In this context, RPA refers to the use of the Kohn‐Sham eigenvalues and wavefunctions in an independent particle approximation. These calculations employed 9 × 9 × 3 *k*‐point sampling with a Gaussian broadening of 0.1 eV, using an arbitrary *k*‐shift and the velocity operator. For the spectra, 400 occupied states and 30 unoccupied states were used for (R/S‐FMBA)_2_PbBr_4_, while 188 occupied states and 30 unoccupied states were used for (FBA)_2_PbBr_4_.

The RPA optical absorption spectra as a function of light frequency *ω* were obtained via the following equation, implemented in the BerkeleyGW code,^[^ ^27^ ^]^

(1) $$\varepsilon_{2}(\omega )=\frac{16\pi^{2}e^{2}}{\hbar^{2}\omega^{2}}\sum_{vck}|e\cdot \left\langle vk|v|ck\right\rangle {|}^{2}\;\delta \;(\hbar \omega -E_{ck}^{\text{DFT}}+E_{vk}^{\text{DFT}})$$

where **e** is the electric‐field polarization vector of the light and ⟨vk|v|ck⟩ is the velocity matrix element, which quantifies the transition probability between the valence band *v* and conduction band *c* at a certain *k*‐point. $E_{ck}^{\text{DFT}}$ and $E_{vk}^{\text{DFT}}$ are the DFT energy eigenvalues of the conduction band and valence bands.

For (R/S‐FMBA)_2_PbBr_4_, the calculations were performed separately along the three Cartesian directions (*x*, *y*, and *z*), where the *z*‐axis is parallel to the *c* crystallographic direction and perpendicular to the inorganic 2D layers, to obtain the polarization‐resolved $\varepsilon_{2}(\omega )$ data. An isotropic (unpolarized) response was subsequently obtained by averaging over the three directions,

(2) $$\varepsilon_{2,\text{unpolarized}}(\omega )=\frac{1}{3}\left[\varepsilon_{2,xx}(\omega )+\varepsilon_{2,yy}(\omega )+\varepsilon_{2,zz}(\omega )\right]$$

For (FBA)_2_Br_4_, the polarizations used instead are the 3 lattice vectors, where *a* and *b* are in the *xy* plane of the inorganic 2D layer and *c* is close to, but slightly off, the *z*‐axis normal to the layer. From the dielectric function, the extinction coefficient k(ω) was calculated as^[^ ^28^ ^]^

(3) $$k(\omega )=\sqrt{\frac{\sqrt{\varepsilon_{1}^{2}(\omega )+\varepsilon_{2}^{2}(\omega )}-\varepsilon_{1}(\omega )}{2}}$$

The absorption coefficient α(ω) was then determined^[^ ^28^ ^]^ via

(4) $$\alpha (\omega )=\frac{2\omega }{c}\;k(\omega )$$

where *c* is the speed of light in vacuum. For comparison to experiment, spectra were plotted in terms of the wavelength λ=hc/E.

### 2.3 Determination of Octahedral Distortion Parameters

**Figure 2:**
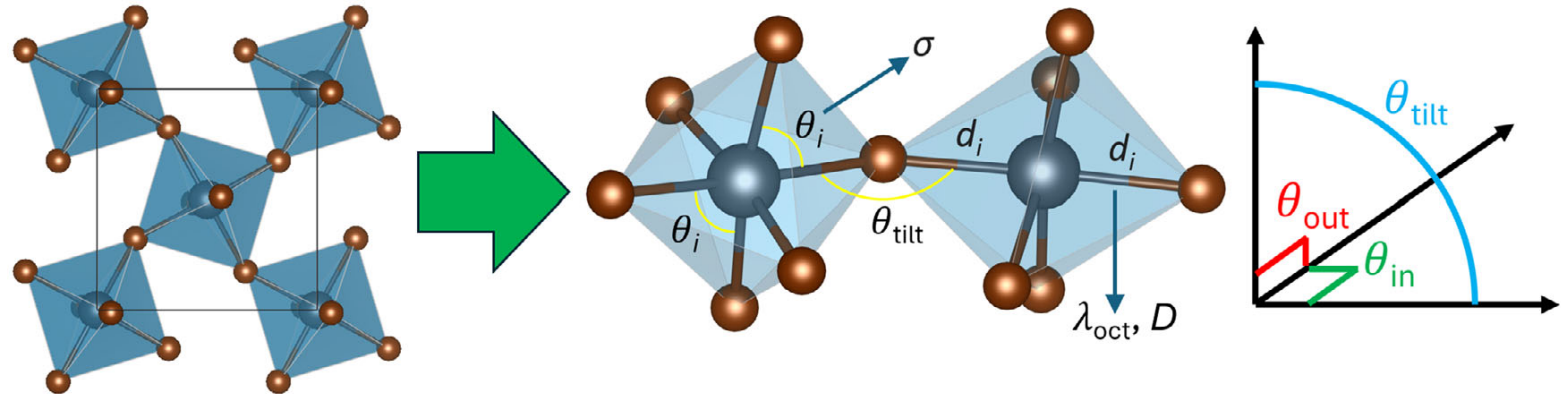
A schematic of octahedral distortion parameters in a 2D perovskite: within an octahedron, deviations *λ*_oct_ and *D* of the bond lengths *d*_*i*_, and deviation *σ* of bond angles *θ*_*i*_; between neighboring octahedra, the tilt angle *θ*_tilt_ and its resolution into components in plane *θ*_in_ and out of plane *θ*_out_, with reference to the 2D perovskite layers.^[^ ^12^ ^]^

The octahedral distortion parameters were analyzed to understand the structural variations in (FBA)_2_PbBr_4_ and (R/S‐FMBA)_2_PbBr_4_. The tilting of the equatorial Pb—Br—Pb bond angles (*θ*_tilt_, and also referred to as *β* in some literature^[^ ^13^, ^29^ ^]^) can be divided into an in‐plane angle (*θ*_in_) and an out‐of‐plane angle (*θ*_out_)^[^ ^12^ ^]^ (**Figure** **2**). To quantify the distortion of the PbBr_6_ octahedra, three primary parameters, *D*_tilt_, *D*_in_, and *D*_out_, were computed. These are defined as *D*_tilt_ =  180° − *θ*_tilt_, $D_{\text{in}}=180{}^{\circ}-\theta_{\text{in}}$, and *D*_out_ =  180° − *θ*_out_. Simpler structures such as (FBA)_2_PbBr_4_ have only a single tilt angle, but there are two angles in our chiral structures since there are inequivalent pairs of octahedra in the structure^[^ ^12^, ^13^ ^]^ and in some cases, there can be even more distinct angles.^[^ ^29^ ^]^ The difference between these angles is defined as $\Delta D_{\text{tilt}}$ (called Δ*β* in refs. [13, 29]).

The bond length distortion indices were calculated as $\lambda_{\text{oct}}=\frac{1}{6}\sum_{i=1}^{6}{\left[\frac{d_{i}-d_{0}}{d_{0}}\right]}^{2}$ and $D=\frac{1}{6}\sum_{i=1}^{6}\frac{\left|d_{i}-d_{0}\right|}{d_{0}}$, where *d*_0_ is the mean Pb—Br bond length and *d*_*i*_ represents individual bond distances. These metrics differ only in the exponent, where *λ*_oct_ is the fractional variance, whereas *D* is the fractional mean difference from the average. The bond angle variance, $\sigma^{2}=\frac{1}{11}\sum_{i=1}^{12}{\left(\theta_{i}-90{}^{\circ}\right)}^{2}$, was used to measure the deviation from ideal octahedral geometry, with *θ*_*i*_ denoting the Br—Pb—Br bond angles. These metrics correspond to a given octahedron, and thus, there may be multiple different values if there are multiple metal centers in the unit cell. Each of the structures considered in this work has 4 Pb‐centered octahedra per unit cell, but they are related by symmetry and therefore have identical parameters.

The calculated octahedral distortion parameters (*λ*_oct_, *D*, *σ* ^2^, *D*_tilt_, *D*_out_, *D*_in_) were computed with a single Python code, which provides a quantitative insight into the structural differences between the experimental (XRD) and theoretical (DFT) results for (FBA)_2_PbBr_4_ and (R/S‐FMBA)_2_PbBr_4_. The code was adapted and extended from the MATLAB code provided in ref. [12], and carefully benchmarked against the results from that code as well as the published results. The Python code analysis provides an efficient approach to extract these parameters systematically, which can further link to their structural, electronic, and optical properties. The code is provided in the Supplementary Information, along with a file of the input coordinates used to run the code on each of our structures. The code requires atomic positions to be provided in Cartesian (Ångstrom) units. Appropriate coordinates can be determined by inspection of the crystal structure with visualization software such as VESTA^[^ ^18^ ^]^ or XCrySDen.^[^ ^30^ ^]^

Given two octahedra centered on atoms Pb1 and Pb2, *D*_tilt_ is calculated as the angle between the Pb1—Br and Br—Pb2 bond vectors. The code uses two distinct approaches to compute *θ*_in_ and *θ*_out_: a projection with respect to a plane defined by 3 Pb atoms, as defined in ref. [12], and a projection with respect to the *z*‐direction (often the same as the (001) plane perpendicular to the crystal *c*‐axis, as used for projections in ref. [13]). For the Pb atoms projection approach, *D*_in_ is determined by projecting the Br atom onto the plane defined by Pb1, Pb2, and an atom Pb3 in an adjacent octahedron in the same layer (which may be a periodic image of Pb1 or Pb2), and calculating the angle within this plane. *D*_out_ involves projecting the Br atom onto a plane orthogonal to the Pb1—Pb2—Pb3 plane. The angle between Pb1 and Pb2 within this orthogonal plane is then used to calculate *D*_out_. The code also implements, alternatively, a projection into a plane defined by an arbitrary normal vector. In the case of our 2D layered perovskites, two logical choices of normal are the *c*‐axis, which is the direction of interlayer periodicity,^[^ ^13^ ^]^ or the direction perpendicular to the layers (which is *z* in our coordinate system, in which case the projection is into the *xy* plane). For orthorhombic (R/S‐FMBA)_2_PbBr_4_, the *c*‐axis is perpendicular to the layers, so the two choices coincide. For monoclinic (FBA)_2_PbBr_4_, they are different, and we will choose the perpendicular *z*‐axis as the normal. We will show results from both the 3‐atom plane and the *z*‐axis. The *z*‐axis approach results are denoted $D_{\text{out}}^{z}$ and $D_{\text{in}}^{z}$ in this work. This method often gives quite similar results to the 3D projection method. The *z*‐axis approach may be more suitable in cases of significant corrugation in the perovskite layer,^[^ ^13^ ^]^ in which case choice of different atoms, Pb3, could give differing results. On the other hand, use of a plane of 3 atoms is more suitable if the *c*‐axis is not close to perpendicular to the inorganic layers, and a direction perpendicular to the layer cannot be easily defined.

## 3 Results and Discussion

### 3.1 Overall Structure

**Figure 3:**
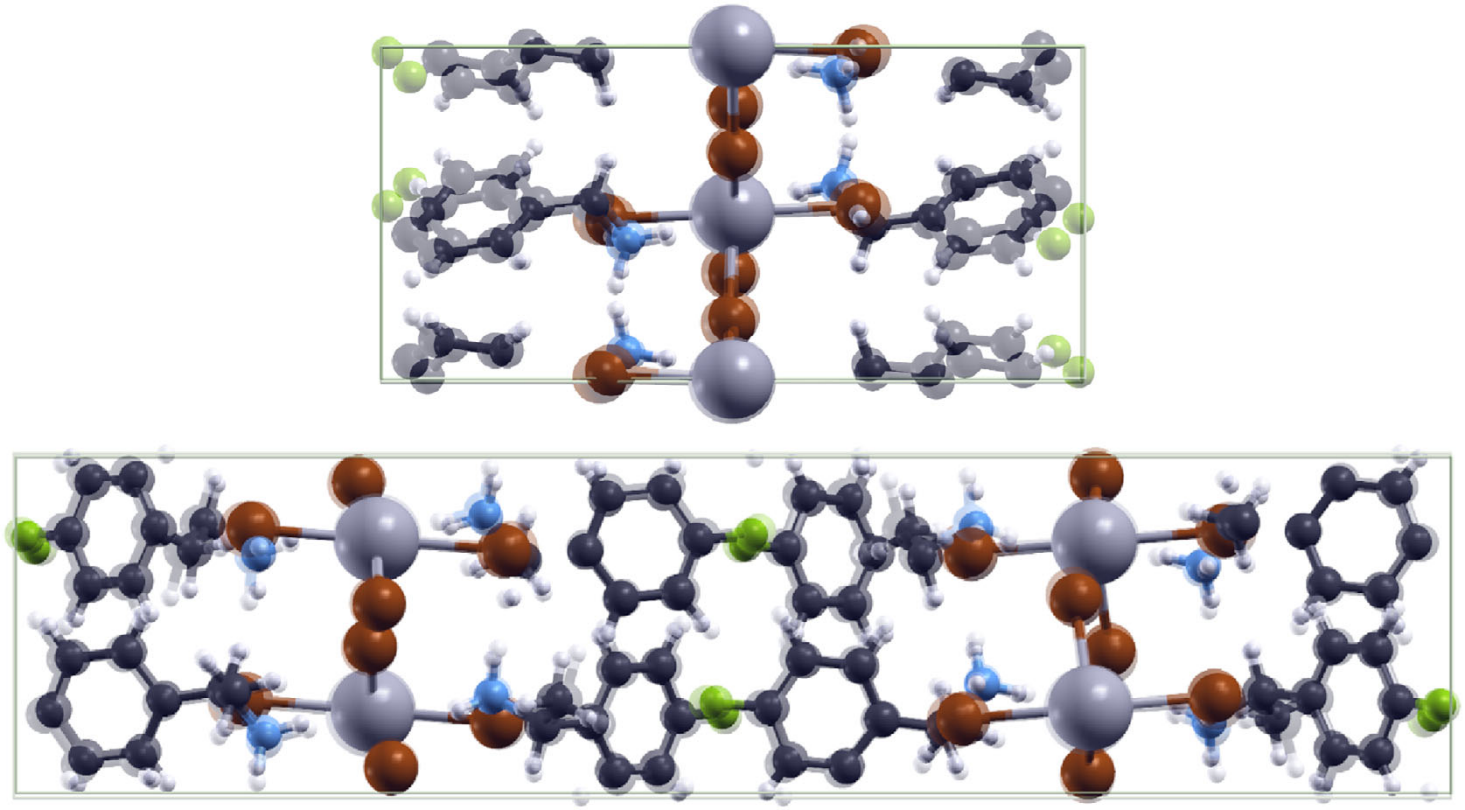
Comparison of XRD (solid) and DFT‐relaxed (partially transparent) structures of (FBA)_2_PbBr_4_ (top) and (R‐FMBA)_2_PbBr_4_ (bottom).

DFT‐relaxed structures were compared to structures determined by XRD to assess the accuracy of computational methods (**Figure** **3**). For (FBA)_2_PbBr_4_, the DFT‐relaxed and XRD structures appeared similar, but the benzene rings were rotated more parallel to the *xz* plane in the DFT calculation. This effect was much more pronounced when van der Waals corrections were not included. It is also observed that the DFT‐relaxed lattice parameters are closer to the XRD structure when van der Waals corrections are used (Table 1). This motivated us to use the van der Waals corrections for further calculations on (R/S‐FMBA)_2_PbBr_4_. Similarly, the (R/S‐FMBA)_2_PbBr_4_ relaxed and XRD structures were very similar except for slight rotations of the benzene ring and small displacements of other atoms.

Given the experimental synthesis, we expect the R‐FMBA and S‐FMBA structures to be enantiomers. The structures relaxed by DFT from the XRD coordinates were compared by reflecting the R‐FMBA structure in the *yz* plane and looking at differences in coordinates. The maximum displacement between the atoms in the two structures was on the order of 0.02 Å. We ascribe any difference to small irreproducibility or noise in the synthesis and XRD, since any true difference in crystal structure is not possible by symmetry. Some further results are presented only for one enantiomer, since in this work, all the properties we calculate are achiral and would be identical for exact enantiomers. The small differences in properties between R‐FMBA and S‐FMBA in our calculations are due only to small structural differences.

### 3.2 Lattice Parameters

Lattice parameters from DFT were compared to those obtained experimentally by XRD (Table 1). The relaxed lattice parameters for (FBA)_2_PbBr_4_ obtained with the PBE functional with Grimme‐D2 van der Waals (vdW) corrections were in good agreement with the XRD parameters, particularly the angle *β*. Calculations without vdW corrections yielded lattice parameters that deviated further from the experimental values, confirming the importance of including dispersion corrections for layered perovskite systems. The deviations are largest for the *c* parameter because the interlayer spacing is controlled by challenging‐to‐describe vdW interactions, whereas the *a* and *b* parameters are controlled primarily by stronger in‐plane bonding interactions, which are more accurate in DFT approximations. For (R/S‐FMBA)_2_PbBr_4_, the PBE functional with vdW corrections gave relaxed lattice parameters that were very close to those from XRD, as shown in Table 1. Based on these results, all subsequent calculations used the PBE functional with van der Waals corrections. We also find that there is close agreement between our XRD and the literature^[^ ^7^ ^]^ (Table 1).

### 3.3 Octahedral Distortion Parameters

**Figure 4:**
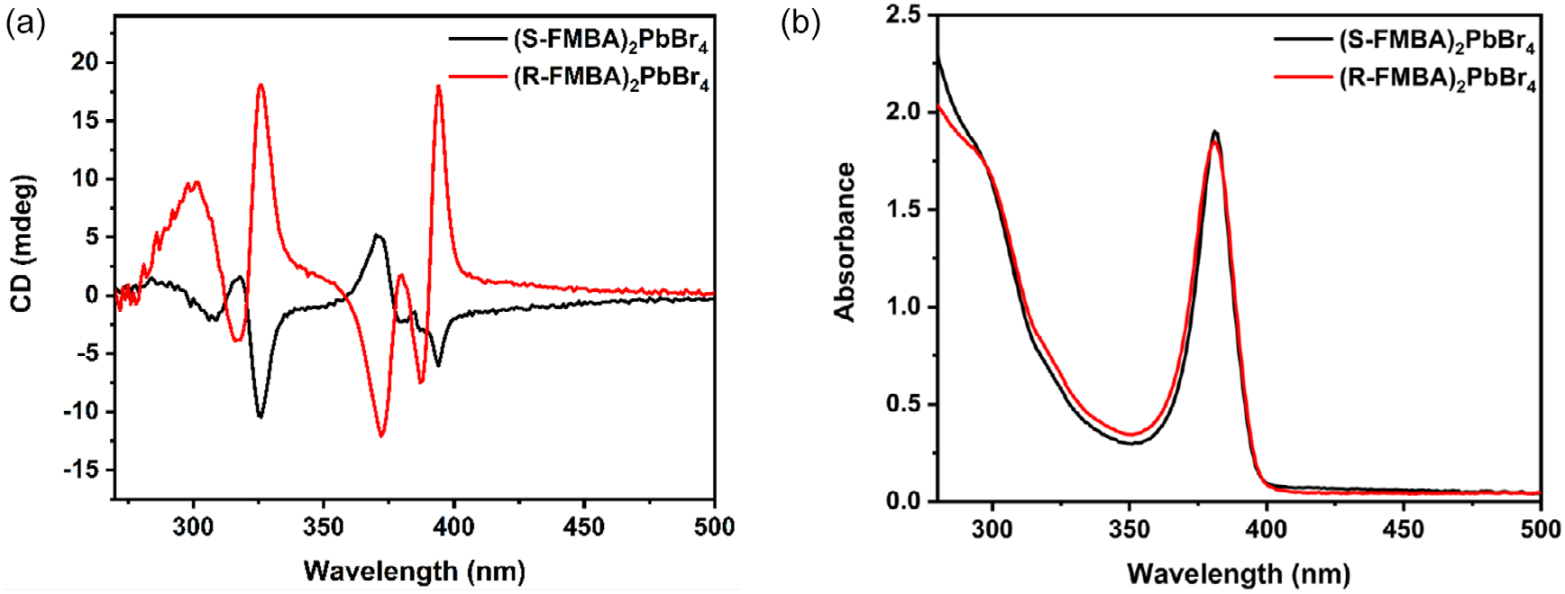
Measured spectra of (R/S‐FMBA)_2_PbBr_4_: a) circular dichroism, and b) absorbance.

The results are presented in **Table** **2**. We notice good agreement between our XRD and the literature XRD^[^ ^7^ ^]^ across the parameters, for both angles. There are some small differences between DFT and XRD values. The distortion angles *D*_tilt_, *D*_out_, and *D*_in_, as well as the $\Delta D_{\text{tilt}}$, are somewhat overestimated in DFT. We note very large values of $\Delta D_{\text{tilt}}$ for FMBA, around 14°–15° from the XRD structure (both in this work and literature), which are among the highest reported. For comparison, ref. [13] reported 3 compounds with 14°–15°, and ref. [29] reported a compound with 11°. This parameter shows a very strong inversion symmetry‐breaking by the octahedral distortions, revealing the cause of the bright circularly polarized emission that was observed,^[^ ^7^ ^]^ as well as our circular dichroism measurements (**Figure** **4**a). The values of $\Delta D_{\text{in}}$, also found in ref. [13] to correlate with spin splittings, are similar and slightly smaller. By contrast, achiral FBA has $\Delta D_{\text{tilt}}=0$.

**Table 2:** Comparison of structural parameters for (FBA)_2_PbBr_4_ and (R/S‐FMBA)_2_PbBr_4_ from XRD and DFT (PBE + vdW), except one comparison to PBE alone (no vdW) with results from this work except XRD values from literature,^[^ ^7^ ^]^ as marked with *; we computed the others from their reported structures.

Compound	Method	*λ*_oct_ (×10^−3^)	*D* (×10^−2^)	*σ* ^2^ [°]^2^	*D*_tilt_ [°]	*D*_out_ [°]	*D*_in_ [°]	Δ*D*_tilt_[°]	$D_{\text{out}}^{z}$ [°]	$D_{\text{in}}^{z}$ [°]
(FBA)_2_PbBr_4_	XRD	0.02	0.5	5.7	30.2	0.8	30.2	0	4.6	30.1
	DFT	0.1	0.9	7.3	33.0	3.5	32.9	0	3.5	32.9
	DFT, no vdW	0.0	0.16	9.4	32.7	1.2	32.6	0	1.2	32.6
(R‐FMBA)_2_PbBr_4_	XRD^[^ ^7^ ^]^	1.13^*^	3.0	50.5^*^	37.7, 23.0	14.9, 4.0	35.0, 22.7	14.7	15.4, 3.7	34.9, 22.7
	XRD	1.3	3.1	48.6	37.0, 22.5	14.2, 4.2	34.5, 22.2	14.5	14.7, 4.0	34.3, 22.2
	DFT	0.6	1.7	61.8	41.0, 25.3	14.9, 4.3	38.6, 24.9	15.7	15.9, 3.6	38.3, 25.0
(S‐FMBA)_2_PbBr_4_	XRD^[^ ^7^ ^]^	1.12^*^	3.0	50.7^*^	37.7, 22.9	14.9, 4.1	35.0, 22.5	14.8	15.3, 3.8	34.8, 22.6
	XRD	1.3	3.1	48.2	36.9, 22.6	14.2, 4.2	34.4, 22.2	14.3	14.6, 4.0	34.3, 22.3
	DFT	0.6	1.8	61.8	41.0, 24.9	14.5, 5.3	38.7, 24.3	16.1	15.6, 4.6	38.3, 24.5

(R/S‐FMBA)_2_PbBr_4_ has slightly larger *D*_tilt_ than (FBA)_2_PbBr_4_. All 3 structures show distortions that are predominantly in plane rather than out of plane. The larger of the two *D*_out_ angles for (R,S‐FMBA)_2_PbBr_4_ is significantly larger than those for (FBA)_2_PbBr_4_, indicating greater distortions induced by the larger chiral organic cations. The two *D*_in_ angles for FMBA are similar on average to FBA but split by 12°–14°.

We further compared the octahedral distortion parameters obtained from DFT calculations of (FBA)_2_PbBr_4_ with and without vdW correction. As summarized in Table 2, bond‐related and angle‐related parameters are similar in both cases, despite more substantial deviations in lattice parameters.

An additional comparison to make is the Pb‐plane projection (*D*_out_, *D*_in_) vs. the *z*‐axis projection ($D_{\text{out}}^{z}$, $D_{\text{in}}^{z}$). We find very similar results between the two methods. The FBA DFT structures have identical values from the two projection methods since the Pb atoms in fact lie in the *xy* plane. Larger differences between the two approaches can arise in other cases with larger corrugation of the layers. Further studies of correlation of these octahedral tilting parameters with optoelectronic properties can help establish which approach may be more useful in physical understanding and crystal design.

### 3.4 Optical Properties

The circular dichroism spectrum was measured to confirm chirality, which is shown in Figure 4 with comparison to the absorbance of each enantiomer. Differences from exactly mirrored spectra are similar to those seen for similar materials,^[^ ^31^, ^32^ ^]^ which can arise from subtle differences between films.^[^ ^33^ ^]^

**Figure 5:**
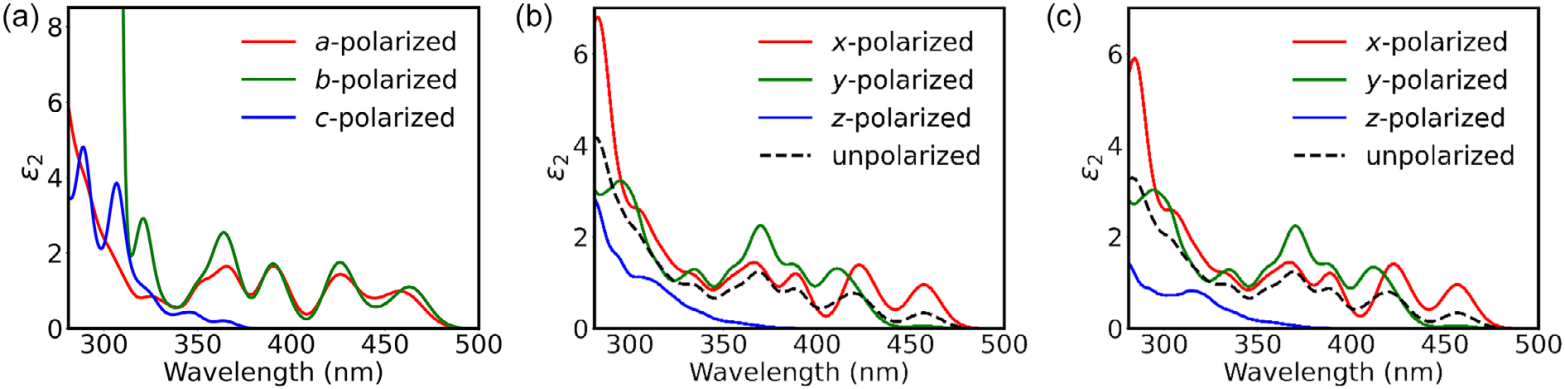
Polarization‐dependent optical absorption spectra, calculated with RPA, for a) (FBA)_2_PbBr_4_, b) (R‐FMBA)_2_PbBr_4_, and c) (S‐FMBA)_2_PbBr_4_ structures.

The polarized optical absorption spectra for FBA_2_PbBr_4_ and (R/S‐FMBA)_2_PbBr_4_ were calculated (**Figure** **5**). In each case, the long‐wavelength onset is due to in‐plane‐polarized transitions, with *c*/*z*‐polarized transitions contributing only below around 350 nm. Due to quantum confinement in the 2D layers, only in‐plane transitions of the layer appear at low energy, and as we will see in the next section, the *c*/*z*‐polarized transitions involve the organic cations. In FMBA, the onset is dominated by *y*‐polarization, and the *x*‐polarized transitions have very low intensity at the onset. This greater in‐plane anisotropy than FBA can be attributed to the greater difference between the *a* and *b* lattice parameters in FMBA (7.88 and 8.82 Å, vs. FBA's 8.14 and 8.03 Å, in DFT), as caused by the distortion due to the larger FMBA cations in the crystal. The strong out‐of‐plane and lesser in‐plane optical anisotropy of these 2D perovskites could be used with orientation of crystals in device applications to control and optimize light absorption and emission.

**Figure 6:**
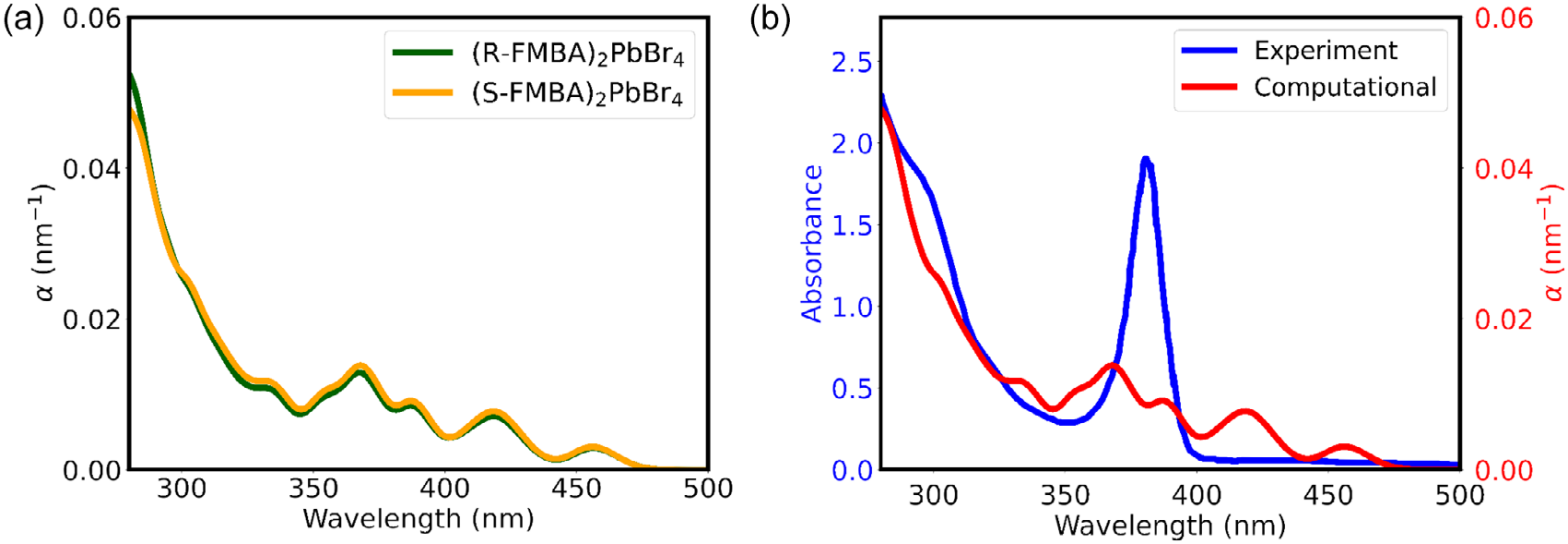
a) Computed absorption coefficients for (R/S‐FMBA)_2_PbBr_4_, and b) computed and measured absorption spectra for (S‐FMBA)_2_PbBr_4_.

Comparing R and S structures of FMBA, only slight differences are found in the absorption coefficients (**Figure** **6**a). We compared the experimental spectrum in Figure 6b. The experimental spectrum displays a prominent peak at 381 nm, comparable to the peak around 375 nm previously reported,^[^ ^7^ ^]^ and a large rise around 300 nm. By contrast, the computed spectra show an onset at a longer wavelength around 475 nm with several small peaks, but also have the large rise around 300 nm. The onset corresponds to transitions around the bandgap (see below, Figure 8). The disagreement in the onset and lack of well‐defined peak is because the DFT‐RPA level of theory used in these calculations cannot describe excitonic peaks,^[^ ^27^, ^34^, ^35^ ^]^ and also due to the lack of quasiparticle renormalization and neglect of SOC. At a higher level of theory, electron‐electron interactions would increase the energy, and electron‐hole interactions would bring the transitions together into a sharper excitonic peak.^[^ ^26^ ^]^

### 3.5 Partial Density of States (PDOS)

**Figure 7:**
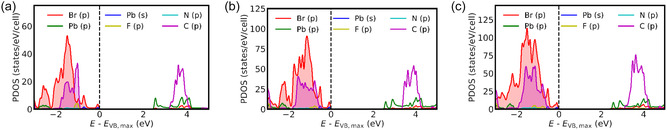
PDOS showing major orbital contributions near valence and conduction band edges in a) (FBA)_2_PbBr_4_, b) (R‐FMBA)_2_PbBr_4_, and c) (S‐FMBA)_2_PbBr_4_ structures.

PDOS analysis was performed to assess which orbitals contributed the most to the transitions (**Figure** **7**). PDOS analysis for both (FBA)_2_PbBr_4_ and (R‐FMBA)_2_PbBr_4_ showed that the Br p‐orbital contribution near the valence bands was dominant compared to all other orbitals. The second‐highest contribution near the valence bands was from the Pb s‐orbital. The Pb p‐orbital contribution was prominent in the conduction bands closer to the valence bands.

### 3.6 Electronic Structure

**Figure 8:**
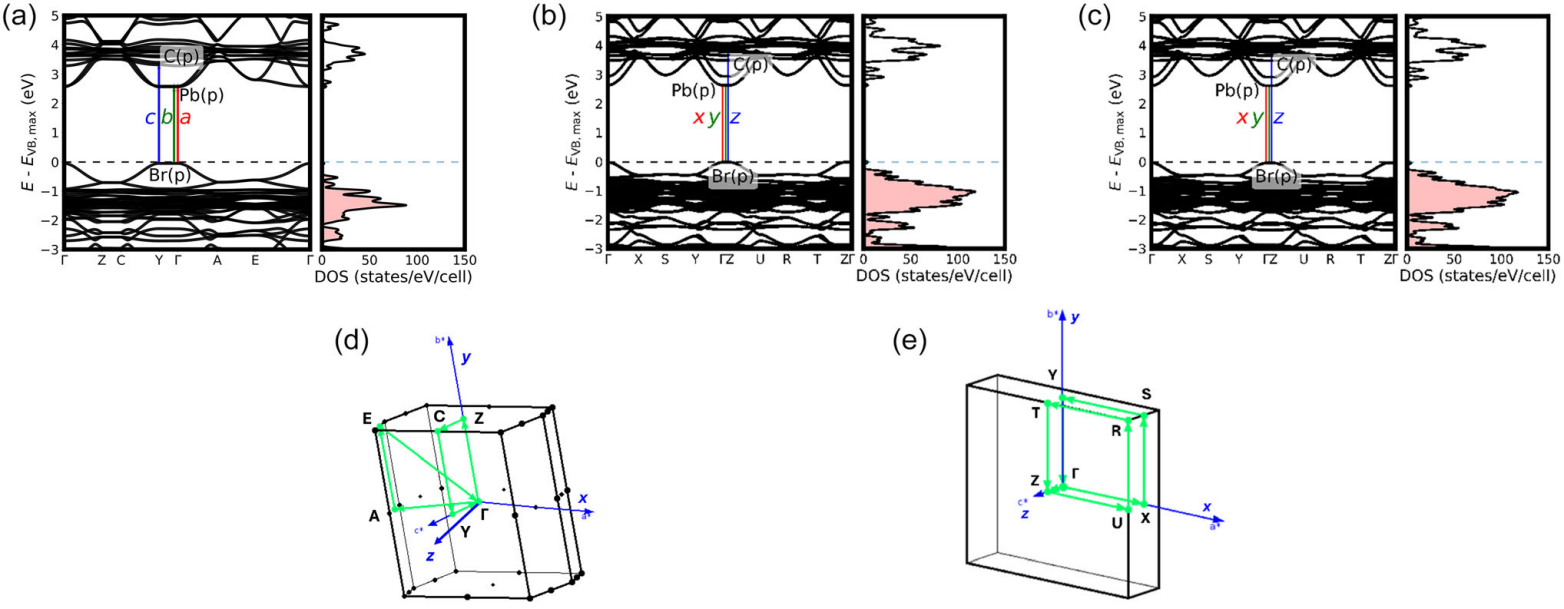
Calculated electronic band structures and corresponding DOS for a) (FBA)_2_PbBr_4_, b) (R‐FMBA)_2_PbBr_4_, and c) (S‐FMBA)_2_PbBr_4_. The lowest‐energy optical transitions are marked for each polarization, and the predominant orbital character is marked for the valence and conduction bands involved in these levels. The Brillouin zone paths are shown below for d) (FBA)_2_PbBr_4_ and e) (R‐FMBA)_2_PbBr_4_.

The electronic band structure and significant orbital contributions to the electronic transitions were calculated for each material, and are shown in **Figure** **8**. A direct bandgap was found in each case. The band gap of (FBA)_2_PbBr_4_ was calculated to be 2.54 eV. The lowest‐energy transitions were due to *a*‐ and *b*‐polarizations, with *b* somewhat more intense. The transitions near the band gap primarily involved the *Γ* and Y points in the Brillouin zone. The in‐planepolarized transitions were predominantly from Br p orbitals to Pb p orbitals, whereas *c*‐polarized transitions were predominantly from Br p to C p. (R/S‐FMBA)_2_PbBr_4_ had a slightly larger calculated band gap of 2.65 eV. The lowest‐energy transitions were similarly due to *x‐* and *y*‐polarizations near the *Γ* point, though with very low intensity for *x* near the onset, and the main orbital contributions to the transitions are the same as in FBA. A summary of the electronic bandgaps and the electronic transitions, from valence bands (VB) to conduction bands (CB), for different polarizations is shown in **Table** **3**.

**Table 3:** Lowest‐energy optical transitions for each polarization, with corresponding conduction band (CB) and valence band (VB) indices (counting starts with 1 at the band edge) and their principal atomic orbital contributions, and predominant *k*‐point in the band structure, for (FBA)_2_PbBr_4_ and (R/S‐FMBA)_2_PbBr_4_.

Material	*E*_g_ [eV]	Polarization	CB	CB orb.	VB	VB orb.	*k*‐point
(FBA)_2_PbBr_4_	2.54	*a*	1	Pb p	1	Br p	*Γ*
	2.54	*b*	1	Pb p	1	Br p	*Γ*
	3.30	*c*	3	C p	1	Br p	Y
(R‐FMBA)_2_PbBr_4_	2.65	*x*	1	Pb p	2	Br p	*Γ*
	2.65	*y*	1	Pb p	1	Br p	*Γ*
	3.79	*z*	10	C p	1	Br p	*Γ*
(S‐FMBA)_2_PbBr_4_	2.65	*x*	1	Pb p	2	Br p	*Γ*
	2.65	*y*	1	Pb p	1	Br p	*Γ*
	3.75	*z*	10	C p	1	Br p	*Γ*

**Figure 9:**
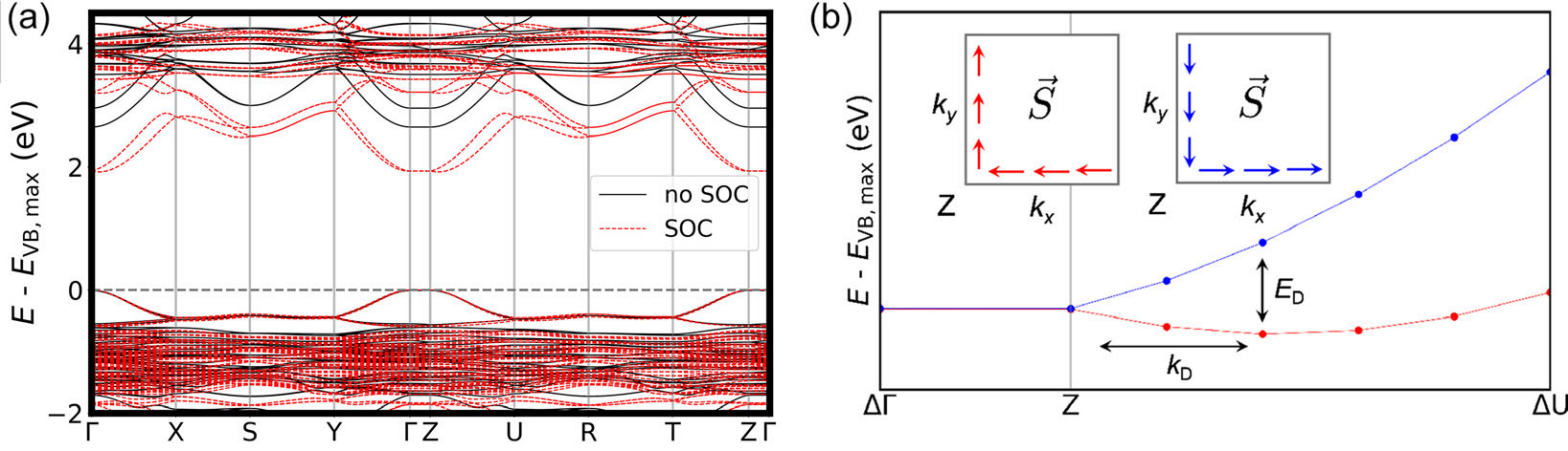
a) Band structure calculated with and without SOC for (R‐FMBA)_2_PbBr_4_. b) Conduction band minimum dispersion around Z, showing spin splitting. Insets: schematic of spin texture of outer (red) and inner (blue) bands, showing the Dresselhaus form in the Z‐U‐T plane.

We estimated the spin splitting of (R‐FMBA)_2_PbBr_4_, which is due to SOC but also inversion symmetry‐breaking. The band structure with SOC (having a gap at k=Γ reduced to 1.9 eV) is shown in **Figure** **9**a. Splitting in the top of the valence band is not resolvable, but can be found at the conduction band minimum near the Z point. We calculated the spin texture ⟨S⟩ around *Z* and found that in the *k*_*x*_, *k*_*y*_ plane, ⟨S⟩ is parallel to Δk (with values $<S_{x}>\;\approx -0.02$, $<S_{y}>\;\approx 0.14$). This pattern is characteristic of the Dresselhaus‐type splitting as found in some hybrid perovskites,^[^ ^36^ ^]^ along with varying <*S*_*z*_> components (Figure 9b). We find energy splitting $E_{D}=51\;\text{meV}$ and *k*‐shift $k_{D}=0.04{\text{ Å}}^{-1}$ in the direction of the U point, leading to Dresselhaus parameter $a=E_{D}/2k_{D}=0.8\;\text{eV Å}$. For comparison, in another chiral 2D perovskite with Δ*D*_tilt_ =  11.9°, values $E_{R}=75\;\text{meV}$, $k_{R}=0.02{\text{ Å}}^{-1}$, and $a=E_{R}/2k_{R}=2.21\;\text{eV Å}$ were found, thus having a larger spin‐splitting parameter *a* though a smaller *D*_tilt_.

## 4 Conclusion

We employed DFT to investigate the structural and octahedral distortion parameters of 2D chiral hybrid perovskites, specifically (FBA)_2_PbBr_4_ and (R/S‐FMBA)_2_PbBr_4_, in conjunction with experimental synthesis and characterization with XRD and UV/vis spectroscopy. The calculations were performed with and without van der Waals corrections and were systematically compared with experimental data obtained from XRD. The results demonstrate that van der Waals corrections improve the accuracy of DFT‐PBE structures for these 3 materials, with reduced discrepancies in lattice parameters. Further studies of the accuracy of octahedral parameters from DFT for other hybrid perovskites would be desirable to assess the generality of these conclusions.

Key distortion parameters within octahedra as well as tilt angles between octahedra (*D*_tilt_, *D*_out_, and *D*_in_), were analyzed. We find small discrepancies between our XRD and previous XRD results,^[^ ^7^ ^]^ indicating the sensitivity of these quantities. We found a very large value of the difference Δ*D*_tilt_ for FMBA, which is associated with chiral symmetry‐breaking and helps to explain the strongly circularly polarized emission that has been observed in this work and the literature.^[^ ^7^ ^]^ A large Dresselhaus spin‐splitting and *a* parameter was found, indicating potential of these materials for spintronic applications.^[^ ^13^ ^]^

Calculated polarized optical spectra showed that low‐energy transitions are polarized in plane, and are Br p to Pb p predominantly, whereas at higher energies *c*/*z*‐polarized Br p to C p transitions are found. There is a significant excitonic peak in experiment which was not captured in our RPA calculations.

We provide a Python code that we developed to facilitate calculations of octahedral distortion parameters in perovskites, to enable investigations of octahedral distortions in broader classes of perovskites and advance our understanding of structure‐property relations in chiral and layered organic–inorganic perovskites.

## Conflict of Interest

The authors declare no conflict of interest.

## Supporting information

Supplementary Material

## Data Availability

The data that support the findings of this study are available in the supplementary material of this article.
