# Supplementary material for: Computational and Experimental Investigation of Chiral and Achiral Two‐Dimensional Organic Lead Bromide Perovskites: Octahedral Distortions and Electronic and Optical Properties
Source: Chemphyschem. 2025 Oct 27;26(24):e202500423. doi: 10.1002/cphc.202500423 (PMC12710165; doi:10.1002/cphc.202500423)
Supplement: Supplementary file 1 — Supplementary Material [file CPHC-26-e202500423-s001.zip › cphc70166-sup-0001-SuppData-S1.pdf]

Supplementary Information: Computational and Experimental Investigation of Chiral and Achiral 2D Organic Lead Bromide Perovskites: Octahedral Distortions and Electronic and Optical Properties

Md Mehdi Masud,[a] Jarek Viera,[b] Azza Ben-Akacha,[b] Biwu Ma,*[b] David A. Strubbe*[a]

[a] Md Mehdi Masud, David A. Strubbe
Department of Physics
University of California, Merced
Merced, California 95343, USA
E-mail: dstrubbe@ucmerced.edu

[b] Jarek Viera, Azza Ben-Akacha, Biwu Ma
Department of Chemistry and Biochemistry
Florida State University
Tallahassee, Florida 32306, USA
E-mail: bma@fsu.edu

Synthesis

Synthesis of $(R\text{-}FMBA)_2PbBr_4$ and $(S\text{-}FMBA)_2PbBr_4$ crystals were obtained by reacting lead oxide and S-/R-FMBA, in a 1:2 stoichiometric ratio, in 6 mL of hydrobromic acid solution. The prepared solutions were then digested at 100°C with 420 rpm for an hour until they became clear solutions and all reagents were dissolved. Then the vials were opened and left to cool down to room temperature at a natural cooling rate. Plate-like transparent crystals were obtained overnight. Crystals were washed with diethyl ether, dried under vacuum, and stored for further use.

Synthesis of $(FBA)_2PbBr_4$ crystals were prepared by dissolving lead oxide and the 4-fluorobenzylamine solution in a 1:1 ratio in a 6 mL hydrobromic acid solution. The solution was then digested at 100°C with 420 rpm for an hour until they became clear solutions and all reagents were dissolved. Then the vial was opened and left to cool down to room temperature at a natural cooling rate. Plate-like transparent crystals were obtained overnight, washed with diethyl ether, dried under vacuum and stored for further use.

Thin films preparation of $(R\text{-}FMBA)_2PbBr_4$, $(S\text{-}FMBA)_2PbBr_4$ 0.2 g/mL DMF solution of dissolved crystals were spin coated on glass substrates in 3000 rpm and annealed at 65°C for 5 minutes.

Structure Determination through X-ray diffraction

Single crystal X-ray crystallography

The single crystal structures of $(FBA)_2PbBr_4$, $(R\text{-}FMBA)_2PbBr_4$, and $(S\text{-}FMBA)_2PbBr_4$ were obtained using Rigaku XtaLAB Synergy-S diffractometer equipped with a HyPix-6000HE Hybrid Photon Counting (HPC) detector and dual Mo and Cu microfocus sealed X-ray sources. The crystals were attached to cryoloop with Paratone-N oil. The data were collected at room temperature. The structures were solved using Olex2 software where XT

refinement package using Least Squares minimization were employed in solving and refining the structures, respectively. Table S1 summarizes the refinement details and the resulting factors. CIF files have been deposited with the CCDC: 2260472 for (R-FMBA)$_2$PbBr$_4$, 2260473 for (S-FMBA)$_2$PbBr$_4$. VESTA was used as the crystal structure visualization software for the images presented in the manuscript.

Powder X-ray Diffraction

PXRD analysis was performed on Rigaku SmartLab powder X-Ray diffractometer with the PhotonMax high-flux 9 kW rotating anode X-ray source coupled with a 1D measurement modes. The diffraction pattern was scanned over the angular range of 3-50 degrees (2θ) with a step size of 0.03 at room temperature.

Table 1. Room temperature crystallographic data for (FBA)$_2$PbBr$_4$, (R-FMBA)$_2$PbBr$_4$, and (S-FMBA)$_2$PbBr$_4$.

**Compound**	(FBA)$_2$PbBr$_4$	(R-FMBA)$_2$PbBr$_4$	(S-FMBA)$_2$PbBr$_4$
Formula	$C_{14}H_{18}Br_4F_2N_2Pb$	$C_{16}H_{22}Br_4F_2N_2Pb$	$C_{16}H_{22}Br_4F_2N_2Pb$
Formula weight/g mol^{-1}	779.13	807.18	807.18
Temperature/K	298.51(10)	296.93(10)	297.5(8)
Crystal system	monoclinic	orthorhombic	orthorhombic
Space group	P2$_1$/c (14)	P2$_1$2$_1$2 (19)	P2$_1$2$_1$2 (19)
a/Å	17.5495(2)	7.88037(11)	7.88175(4)
b/Å	8.14960(10)	8.82621(13)	8.82647(6)
c/Å	8.11350(10)	33.7831(5)	33.8097(18)
α / °	90	90	90
β / °	99.7440(10)	90	90
γ / °	90	90	90
V/Å^3	1143.66(2)	2349.74(6)	2351.95(2)
Z	2	4	4
ρ_{cal}/g cm^{-3}	2.263	2.282	2.280
F(000)	712	1488	1488
2θ range/°	10.23 to 160.44 (0.78 Å)	10.36 to 178.80 (0.77 Å)	10.36 to 154.99 (0.79 Å)
Reflections collected	31481	61323	67076
Independent reflections	2470	4976	4952
R_{int}	0.1583	0.1293	0.0659
R_{sigma}	0.0468	0.0344	0.0201
Data/constraints/parameters	2470/0/98	4976/0/231	4952/0/230
Goodness-of-fit on F^2	1.077	1.104	1.048
Final R indices $\geq 2\sigma$(I)	R_1 = 0.0564, wR_2 = 0.1668	R_1 = 0.0359, wR_2 = 0.0773	R_1 = 0.0221, wR_2 = 0.0623
Final R indices [all data]	R_1 = 0.0569, wR_2 = 0.1680	R_1 = 0.0383, wR_2 = 0.0798	R_1 = 0.0224, wR_2 = 0.0625

Absorption Analysis

Steady-state optical spectra of the crystals were measured at room temperature and 77 K (liquid nitrogen to cool the samples) using a FS5 spectrofluorometer (Edinburgh Instruments).

Circular Dichroism (CD) Measurements

Thin films of the chiral crystals were prepared by dissolving crystals in DMF at a concentration of 0.2 g/mL. The resulting solutions were spin-coated onto clean glass substrates at 3000 rpm, followed by annealing at 65 °C for 5 minutes. The prepared thin films were subsequently used for chiroptical measurements. Circular Dichroism (CD) spectra were recorded using a Chirascan VX instrument from Applied Photophysics. All spectra (CD and absorbance) were recorded from 250-500 nm.
