# Supplementary material for: Computational and Experimental Investigation of Chiral and Achiral Two‐Dimensional Organic Lead Bromide Perovskites: Octahedral Distortions and Electronic and Optical Properties
Source: Chemphyschem. 2025 Oct 27;26(24):e202500423. doi: 10.1002/cphc.202500423 (PMC12710165; doi:10.1002/cphc.202500423)

checkCIF/PLATON report

Structure factors have been supplied for datablock(s) fmbapbbr-sol-1-8_auto

THIS REPORT IS FOR GUIDANCE ONLY. IF USED AS PART OF A REVIEW PROCEDURE FOR PUBLICATION, IT SHOULD NOT REPLACE THE EXPERTISE OF AN EXPERIENCED CRYSTALLOGRAPHIC REFEREE.

No syntax errors found.   CIF dictionary   Interpreting this report

Datablock: fmbapbbr-sol-1-8_auto

Bond precision:	C-C = 0.0154 A		Wavelength=1.54184
Cell:	a=17.5495(2)	b=8.1496(1)	c=8.1135(1)
	alpha=90	beta=99.744(1)	gamma=90
Temperature:	299 K		

	Calculated	Reported
Volume	1143.66(2)	1143.66(2)
Space group	P 21/c	P 1 21/c 1
Hall group	-P 2ybc	-P 2ybc
Moiety formula	Br4 Pb, 2(C7 H9 F N)	Br4 Pb, 2(C7 H9 F N)
Sum formula	C14 H18 Br4 F2 N2 Pb	C14 H18 Br4 F2 N2 Pb
Mr	779.10	779.13
Dx,g cm-3	2.263	2.263
Z	2	2
Mu (mm-1)	22.666	22.666
F000	712.0	712.0
F000'	699.71	
h,k,lmax	22,10,10	22,10,10
Nref	2514	2470
Tmin,Tmax	0.004,0.263	0.001,0.083
Tmin'	0.000	

Correction method= # Reported T Limits: Tmin=0.001 Tmax=0.083
AbsCorr = ANALYTICAL

Data completeness= 0.982   Theta(max)= 80.222

R(reflections)= 0.0564(2371)   wR2(reflections)= 0.1681(2470)

S = 1.077   Npar= 98

The following ALERTS were generated. Each ALERT has the format
test-name_ALERT_alert-type_alert-level.
Click on the hyperlinks for more details of the test.

Alert level C

```
RINTA01_ALERT_3_C  The value of Rint is greater than 0.12
            Rint given   0.158
PLAT241_ALERT_2_C High   'MainMol' Ueq as Compared to Neighbors of        Br1 Check
PLAT241_ALERT_2_C High   'MainMol' Ueq as Compared to Neighbors of         C4 Check
PLAT241_ALERT_2_C High   'MainMol' Ueq as Compared to Neighbors of         C6 Check
PLAT242_ALERT_2_C Low    'MainMol' Ueq as Compared to Neighbors of         C2 Check
PLAT242_ALERT_2_C Low    'MainMol' Ueq as Compared to Neighbors of         C5 Check
PLAT260_ALERT_2_C Large Average Ueq of Residue Including        F1      0.163 Check
PLAT342_ALERT_3_C Low Bond Precision on  C-C Bonds ...............    0.01543 Ang.
PLAT911_ALERT_3_C Missing FCF Refl Between Thmin & STh/L=    0.600         19 Report
                1  4  0,   1  6  0,   2  6  0,   6  0  0,   7  0  0,   8  0  0,
               10  0  0,  11  0  0,  12  0  0,  -5  2  1,  -1  3  1,   0  2  1,
                0  3  1,   1  2  1,   6  1  1,   6  3  1,  -6  2  2,  -1  2  2,
               -2  0  4,
PLAT971_ALERT_2_C Check Calcd Resid. Dens.  0.21Ang From Pb1B           2.41 eA-3
PLAT972_ALERT_2_C Check Calcd Resid. Dens.  1.05Ang From Pb1B          -2.05 eA-3
```

Alert level G

```
PLAT004_ALERT_5_G Polymeric Structure Found with Maximum Dimension          2 Info
PLAT007_ALERT_5_G Number of Unrefined Donor-H Atoms .............           3 Report
             H1A    H1B    H1C
PLAT020_ALERT_3_G The Value of Rint is Greater Than 0.12 .........      0.158 Report
PLAT072_ALERT_2_G SHELXL First  Parameter in WGHT  Unusually Large       0.12 Report
PLAT171_ALERT_4_G The CIF-Embedded .res File Contains EADP Records          1 Report
PLAT232_ALERT_2_G Hirshfeld Test Diff (M-X)  Pb1A     --Br2      .       49.8 s.u.
PLAT232_ALERT_2_G Hirshfeld Test Diff (M-X)  Pb1B     --Br2      .       50.2 s.u.
PLAT232_ALERT_2_G Hirshfeld Test Diff (M-X)  Pb1B     --Br1_b    .        7.3 s.u.
PLAT232_ALERT_2_G Hirshfeld Test Diff (M-X)  Pb1B     --Br2_c    .       49.7 s.u.
PLAT300_ALERT_4_G Atom Site Occupancy of Pb1A       Constrained at       0.84 Check
PLAT300_ALERT_4_G Atom Site Occupancy of Pb1B       Constrained at       0.08 Check
PLAT301_ALERT_3_G Main Residue  Disorder ..............(Resd    1)        32% Note
PLAT779_ALERT_4_G Suspect or Irrelevant (Bond) Angle(s) in CIF ...       9.69 Deg.
              PB1A -BR1  -PB1B    1_555   1_555   1_555 ......     #   27 Check
PLAT779_ALERT_4_G Suspect or Irrelevant (Bond) Angle(s) in CIF ...       0.99 Deg.
              PB1B -BR2  -PB1A    1_555   1_555   1_555 ......     #   28 Check
PLAT789_ALERT_4_G Atoms with Negative _atom_site_disorder_group  #          1 Check
PLAT822_ALERT_4_G CIF-embedded .res Contains Negative PART Numbers          1 Check
PLAT910_ALERT_3_G Missing FCF Reflection(s) Below Theta(Min)[Deg]=       5.11 Note
                1  0  0,
PLAT912_ALERT_4_G Missing # of FCF Reflections Above STh/L=  0.600         24 Note
PLAT913_ALERT_3_G Missing # of Very Strong Reflections in FCF ....          2 Note
                6  0  0,  -1  2  2,
PLAT933_ALERT_2_G Number of HKL-OMIT Records in Embedded .res File         21 Note
               -6  2  2,  -5  2  1,  -2  0  4,  -2  9  5,  -1  2  2,  -1  3  1,
               -1  9  4,   0  2  1,   0  3  1,   1  2  1,   1  4  0,   1  6  0,
                2  6  0,   6  0  0,   6  1  1,   6  3  1,   7  0  0,   8  0  0,
               10  0  0,  11  0  0,  12  0  0,
PLAT969_ALERT_5_G The 'Henn et al.' R-Factor-gap value ..........      4.012 Note
              Predicted wR2: Based on SigI**2  4.19 or SHELX Weight 15.61
```

```
PLAT978_ALERT_2_G Number C-C Bonds with Positive Residual Density.          1 Info
PLAT992_ALERT_5_G Repd & Actual _reflns_number_gt Values Differ by          2 Check
```

```
  0 ALERT level A = Most likely a serious problem - resolve or explain
  0 ALERT level B = A potentially serious problem, consider carefully
 11 ALERT level C = Check. Ensure it is not caused by an omission or oversight
 23 ALERT level G = General information/check it is not something unexpected

  0 ALERT type 1 CIF construction/syntax error, inconsistent or missing data
 15 ALERT type 2 Indicator that the structure model may be wrong or deficient
  7 ALERT type 3 Indicator that the structure quality may be low
  8 ALERT type 4 Improvement, methodology, query or suggestion
  4 ALERT type 5 Informative message, check
```

It is advisable to attempt to resolve as many as possible of the alerts in all categories. Often the minor alerts point to easily fixed oversights, errors and omissions in your CIF or refinement strategy, so attention to these fine details can be worthwhile. In order to resolve some of the more serious problems it may be necessary to carry out additional measurements or structure refinements. However, the purpose of your study may justify the reported deviations and the more serious of these should normally be commented upon in the discussion or experimental section of a paper or in the "special_details" fields of the CIF. checkCIF was carefully designed to identify outliers and unusual parameters, but every test has its limitations and alerts that are not important in a particular case may appear. Conversely, the absence of alerts does not guarantee there are no aspects of the results needing attention. It is up to the individual to critically assess their own results and, if necessary, seek expert advice.

Publication of your CIF in IUCr journals

A basic structural check has been run on your CIF. These basic checks will be run on all CIFs submitted for publication in IUCr journals (*Acta Crystallographica*, *Journal of Applied Crystallography*, *Journal of Synchrotron Radiation*); however, if you intend to submit to *Acta Crystallographica Section C* or *E* or *IUCrData*, you should make sure that full publication checks are run on the final version of your CIF prior to submission.

Publication of your CIF in other journals

Please refer to the *Notes for Authors* of the relevant journal for any special instructions relating to CIF submission.

PLATON version of 04/06/2025; check.def file version of 30/05/2025

Datablock fmbapbbr-sol-1-8_auto - ellipsoid plot

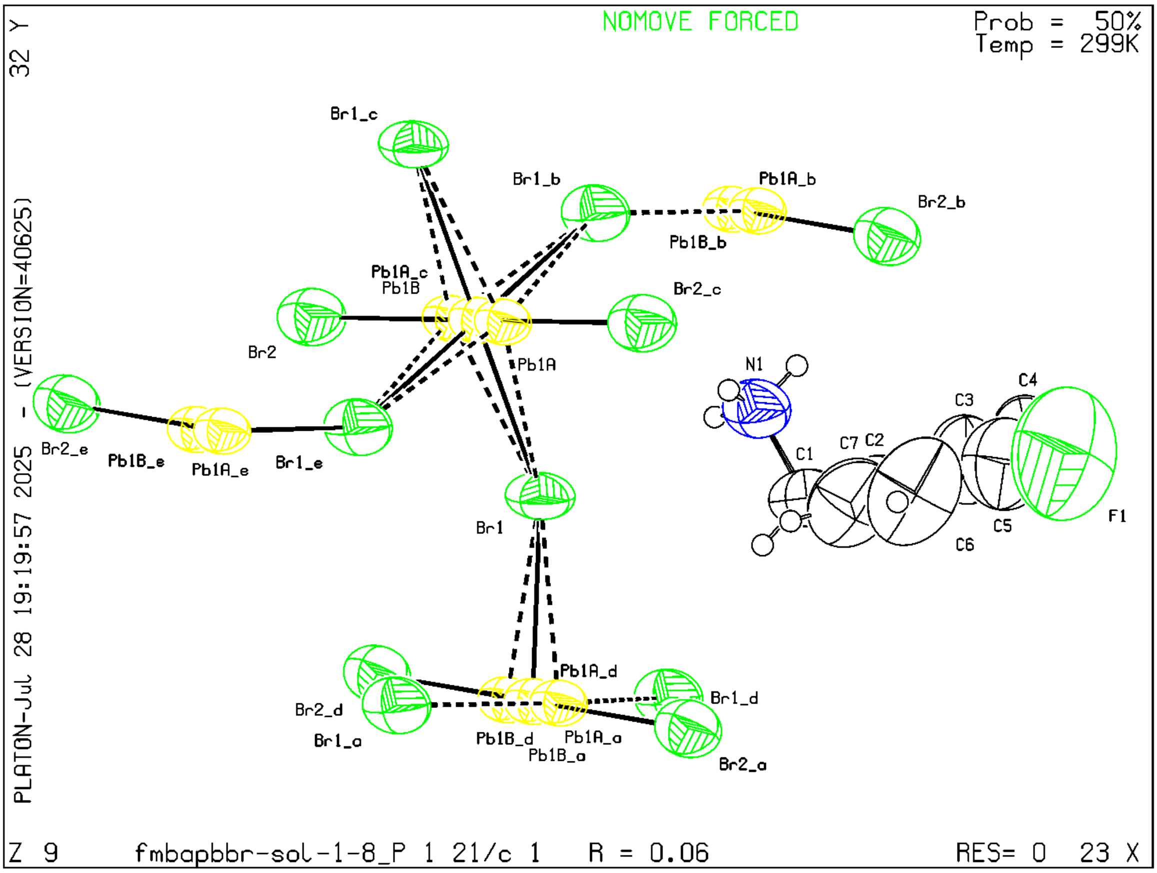

Supplement: Supplementary file 1 — Supplementary Material [file CPHC-26-e202500423-s001.zip › XRD_FBA_refined_checkcif.pdf]
